# The predictive value of laboratory parameters for no‐reflow phenomenon in patients with ST‐elevation myocardial infarction following primary percutaneous coronary intervention: A meta‐analysis

**DOI:** 10.1002/clc.24238

**Published:** 2024-02-23

**Authors:** LinLi Wang, ShuWei Huang, Qiujun Zhou, LiPing Dou, Dongming Lin

**Affiliations:** ^1^ Department of Preventive Medicine, Children's Hospital Zhejiang University School of Medicine, National Clinical Research Center for Child Health Hangzhou China; ^2^ Department of Cardiology The First Affiliated Hospital of Zhejiang Chinese Medical University (Zhejiang Provincial Hospital of Chinese Medicine) Hangzhou China; ^3^ Department of First Clinical Medical College Zhejiang Chinese Medical University Hangzhou China; ^4^ Department of Cardiology The Second Affiliated Hospital of Zhejiang Chinese Medical University Hangzhou China

**Keywords:** meta‐analysis, predictive value, primary PCI, risk factors, ST‐elevation myocardial infarction

## Abstract

To date, the predictive role of laboratory indicators for the phenomenon of no flow is unclear. Hence, our objective was to conduct a meta‐analysis to investigate the association between laboratory parameters and the risk of the no‐reflow phenomenon in patients with ST‐elevation myocardial infarction (STEMI) following primary percutaneous coronary intervention (PCI). This, in turn, aims to offer valuable insights for early clinical prediction of no‐reflow. We searched Pubmed, Embase, and Cochrane Library from the establishment of the database to October 2023. We included case‐control or cohort study that patients with STEMI following primary PCI. We excluded repeated publication, research without full text, incomplete information or inability to conduct data extraction and animal experiments, reviews, and systematic reviews. STATA 15.1 was used to analyze the data. The pooled results indicated that elevated white blood cell (WBC) count (odds ratio [OR] = 1.061, 95% confidence interval [CI]: 1.013–1.112), neutrophil count (OR = 1.324, 95% CI: 1.128–1.553), platelet (PLT) (OR = 1.002, 95% CI: 1.000–1.005), blood glucose (OR = 1.005, 95% CI: 1.002–1.009), creatinine (OR = 1.290, 95% CI: 1.070–1.555), total cholesterol (TC) (OR = 1.022, 95% CI: 1.012–1.032), d‐dimer (OR = 1.002, 95% CI: 1.001–1.004), and fibrinogen (OR = 1.010, 95% CI: 1.005–1.015) were significantly associated with increased risk of no‐reflow. However, elevated hemoglobin was significantly associated with decreased risk of no‐reflow. In conclusion, our comprehensive analysis highlights the predictive potential of various parameters in assessing the risk of no‐reflow among STEMI patients undergoing PCI. Specifically, WBC count, neutrophil count, PLT, blood glucose, hemoglobin, creatinine, TC, d‐dimer, and fibrinogen emerged as significant predictors. This refined risk prediction may guide clinical decision‐making, allowing for more targeted and effective preventive measures to mitigate the occurrence of no‐reflow in this patient population.

## Graphical abstract

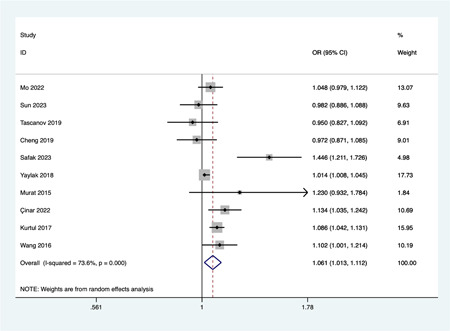

## 1 INTRODUCTION

Primary percutaneous coronary intervention (PCI), pioneered by Grüntzig and colleagues in Switzerland,^1^ has evolved into the gold standard and the preferred treatment for ST‐segment elevation myocardial infarction (STEMI).^2^, ^3^, ^4^ Its widespread adoption over the past 12–15 years has been fueled by its remarkable clinical effectiveness.^5^ The merits of primary PCI were expounded by Rott,^6^ while Goff et al.^7^ demonstrated its superior efficacy, as compared to thrombolytic therapy, in restoring thrombolysis in myocardial infarction (TIMI) flow, consequently leading to a reduction in mortality. Moreover, Singh^8^ proposed that primary PCI is preferable to coronary artery bypass grafting (CABG), despite both procedures yielding similar outcomes in terms of quality of life. However, it is essential to note that favorable outcomes are not always guaranteed with primary PCI, as a commonly reported complication is the occurrence of the no‐reflow phenomenon.^9^, ^10^

The no‐reflow phenomenon is defined as a complex condition characterized by insufficient myocardial perfusion in a coronary artery, even in the absence of apparent evidence of angiographic epicardial vessel obstruction, spasm, or dissection.^11^, ^12^, ^13^ Diagnosis of the no‐reflow phenomenon relies on several diagnostic methods, including angiography, myocardial contrast echocardiography (MCE), and cardiac magnetic resonance imaging (CMRI).^4^ MCE is considered the gold standard for diagnosing no‐reflow, while CMRI is recognized as the most sensitive and specific approach for assessing the extent of no‐reflow.^14^ According to various reports, the incidence of the no‐reflow phenomenon varies, with rates ranging from 2% to 44% among patients undergoing both primary and elective primary PCI. The associated mortality with no‐reflow also exhibits variability, falling within the range of 7.4%–30.3% for all patients experiencing this condition.^15^, ^16^, ^17^ The pathogenesis of the no‐reflow phenomenon is intricate and dynamic, involving factors such as distal atherothrombotic embolization, ischemic injury, reperfusion injury, and an increased susceptibility of coronary microcirculation to damage.^18^, ^19^ The pathogenesis of the no‐reflow phenomenon is intricate and dynamic, involving factors such as distal atherothrombotic embolization, ischemic injury, reperfusion injury, and an increased susceptibility of coronary microcirculation to damage.^20^ Inflammatory mediators induce the expression of adhesion molecules on endothelial cells, promoting leukocyte adhesion and infiltration. This process can lead to microvascular plugging and compromise blood flow. Additionally, Inflammatory responses trigger endothelial cell activation, resulting in capillary endothelial swelling. The increased permeability may contribute to the obstruction of microvessels, exacerbating the no‐reflow phenomenon 37498164.

The occurrence of the no‐reflow phenomenon significantly elevates the risk of adverse clinical outcomes, including mortality, recurrent myocardial infarction (MI), decreased left ventricular ejection fraction (LVEF), left ventricular remodeling, malignant ventricular arrhythmias, heart failure (HF), and cardiac rupture. Given these detrimental effects, accurate detection of no‐reflow, along with the identification of predictive factors, becomes of paramount importance. Currently, the predictive role of laboratory indicators for the no‐reflow phenomenon remains unclear. Therefore, our objective was to conduct a meta‐analysis to investigate the association between laboratory parameters and the risk of the no‐reflow phenomenon in patients with STEMI following primary PCI. The ultimate goal is to provide valuable insights for the early clinical prediction of no‐reflow.

## 2 METHODS

### 2.1 Literature inclusion and exclusion criteria

The inclusion criteria were as follows: the study design is a case‐control or cohort study; patients with STEMI following PCI; studies reporting the risks of the no‐reflow phenomenon; and the language is limited to English.

Exclusion criteria included studies that did not report relevant risk factors, studies focusing exclusively on elderly subjects, duplicate publications, research without full text, incomplete information, or an inability to conduct data extraction, animal experiments, and reviews and systematic reviews.

### 2.2 Search strategy

In this meta‐analysis, we searched Pubmed, Embase, Cochrane Library from establishment of the database to October 2023. The search terms are as follows: (((((((risk factor[Title/Abstract]) OR (risk factors[Title/Abstract])) OR (predictor factor[Title/Abstract])) OR (predictor factors[Title/Abstract])) OR (influence factor[Title/Abstract])) OR (influence factors[Title/Abstract])) OR (predictor[Title/Abstract])) AND ((Percutaneous Coronary Intervention[Title/Abstract]) AND ((no‐reflow[Title/Abstract]))).

### 2.3 Literature screening and data extraction

The literature search, screening, and information extraction were all independently carried out by two researchers. In cases of uncertainty, decisions were reached through discussion or consultation with a third party. Data extraction encompassed author details, publication year, study design, country, sample size, no‐reflow occurrence, demographic information such as sex and age, prevalence of hypertension and diabetes, number of smokers, and odds ratios (ORs) with corresponding 95% confidence intervals (95% CIs) for relevant risk factors.

### 2.4 Literature quality assessment

Two researchers independently conducted quality assessments of the literature using the Newcastle‐Ottawa Scale (NOS)^21^ for cohort and case‐control studies. In cases of discordant opinions, resolution was achieved through discussion or consultation with a third party. The meta‐analysis adhered to the relevant guidelines outlined in the Preferred Reporting Items for Systematic Reviews and Meta‐analysis statement (PRISMA statement).^22^

### 2.5 Data synthesis and statistical analysis

Data analysis was conducted using STATA 15.1 (StataCorp LP)^23^ was used to analyze the data. OR with 95% confidence intervals (95%CI) were utilized for analyzing the risk factors of cardiovascular and cerebrovascular disease subtypes. Heterogeneity was assessed using the *I* ^2^ statistic. If the heterogeneity test yielded a *p* ≥ .1 and *I* ^2^ ≤ 50%, indicating homogeneity among studies, the fixed‐effects model was employed for combined analysis. In cases where *p* < .1 and *I* ^2^ > 50%, suggesting study heterogeneity, sensitivity analysis was performed (each trial was systematically excluded one by one, followed by a combined analysis of the remaining trials) to identify the source of heterogeneity. If substantial heterogeneity persisted, the random‐effects model was considered, or, if necessary, a descriptive analysis without combining results. As there were fewer than five articles for each indicator in the study, no publication bias detection was carried out.

## 3 RESULTS

### 3.1 The results of literature search

In this study, a total of 484 studies were initially identified from the database. Following the removal of duplicate studies, 253 unique studies remained. After reviewing titles and abstracts, 49 publications were excluded, including 28 reviews and systematic reviews, 12 case reports, and 9 animal experiments. Consequently, a total of 204 studies met the inclusion criteria. Finally, 24 articles were included in the meta‐analysis (Figure S1).

### 3.2 Baseline characteristics and quality assessment of the included studies

A total of 20 case‐control and four cohort studies were included in this meta‐analysis, encompassing a cumulative sample size of 14,790 patients, among whom 2575 patients exhibited the no‐reflow phenomenon. Patients from 10 studies originated in China, while the remaining 14 studies involved patients from Turkey. Among all patients, the prevalence of hypertension ranged from 29.63% to 63.97%, diabetes ranged from 16.76% to 37.33%, and the history of smoking varied from 28.63% to 76.35%. The NOS scores used for quality assessment were consistently above 6 (Table 1).

**Table 1:** Baseline characteristics and quality assessment of the included studies.

Reference	Year	Study design	Country	Sample size	Sample size of no‐refow	Age	Sex (male/female)	Hypertension (%)	Diabetes mellitus (%)	Smoking (%)	NOS score
Mo et al.^24^	2022	Cohort	China	674	84	62 ± 16	504/170	53.86	24.78	54.60	7
Şimşek et al.^25^	2021	Cohort	Turkey	905	206	—	741/164	33.92	32.71	76.35	7
Li et al.^26^	2022	Case‐control	China	690	108	60 (52–70)	536/154	55.80	27.68	50.87	8
Yu et al.^27^	2022	Case‐control	China	914	184	59 ± 11	738/176	49.78	25.71	67.18	8
Zhang et al.^28^	2022	Cohort	China	401	78	—	299/102	42.89	23.69	58.85	8
Liu et al.^29^	2022	Case‐control	China	433	80	60 (51–69)	366/77	58.89	22.63	66.97	7
Şahinkus et al.^30^	2016	Case‐control	Turkey	90	44	—	68/22	38.89	20.00	48.89	6
Şenoz et al.^31^	2021	Case‐control	Turkey	247	43	58.1 ± 13.1	179/68	63.97	29.55	37.25	7
Sun et al.^32^	2023	Cohort	China	778	117	—	582/196	53.73	26.22	53.47	9
Liu et al.^33^	2021	Case‐control	China	262	142	—	219/43	51.53	—	28.63	7
Tascanov et al.^34^	2019	Case‐control	Turkey	81	41	—	52/29	29.63	32.10	41.98	8
Dogan et al.^35^	2015	Case‐control	Turkey	173	45	58.4 ± 11.5	140/33	43.35	16.76	68.21	6
Cheng et al.^36^	2019	Case‐control	China	218	39	—	180/38	51.38	29.36	61.93	9
Safak et al.^37^	2023	Case‐control	Turkey	404	103	—	259/145	43.32	18.81	35.64	8
Yaylak et al.^38^	2018	Case‐control	Turkey	3804	471	57.2 ± 11.3	3126/678	39.17	27.29	—	6
Yildirim et al.^39^	2021	Case‐control	Turkey	217	70	65.4 ± 10.6	151/66	29.49	37.33	41.94	9
Dogdus et al.^40^	2020	Case‐control	Turkey	137	45	56.2 ± 11.5	88/49	48.18	29.20	35.04	7
Murat et al.^41^	2015	Case‐control	Turkey	501	91	59.0 ± 13.0	368/133	37.13	28.34	51.50	8
Çinar et al.^42^	2022	Case‐control	Turkey	838	91	—	563/275	48.33	21.00	60.14	9
Abacioglu et al.^43^	2021	Case‐control	Turkey	551	41	60.5 ± 10.8	369/182	51.91	36.30	36.30	7
Zhao et al.^44^	2019	Case‐control	China	510	98	61.1 ± 11.2	392/108	48.43	29.41	57.06	7
Kurtul et al.^45^	2017	Case‐control	Turkey	1206	194	58.7 ± 13.1	908/298	34.99	29.02	53.23	8
Kurtul et al.^46^	2014	Case‐control	Turkey	520	117	—	—	—	—	—	7
Wang et al.^47^	2016	Case‐control	China	236	43	—	192/44	61.44	31.78	46.61	7

Abbreviation: NOS, Newcastle‐Ottawa Scale.

### 3.3 Results of meta‐analysis

#### 3.3.1 Inflammatory cells

##### White blood cell (WBC) count

**Figure 1:**
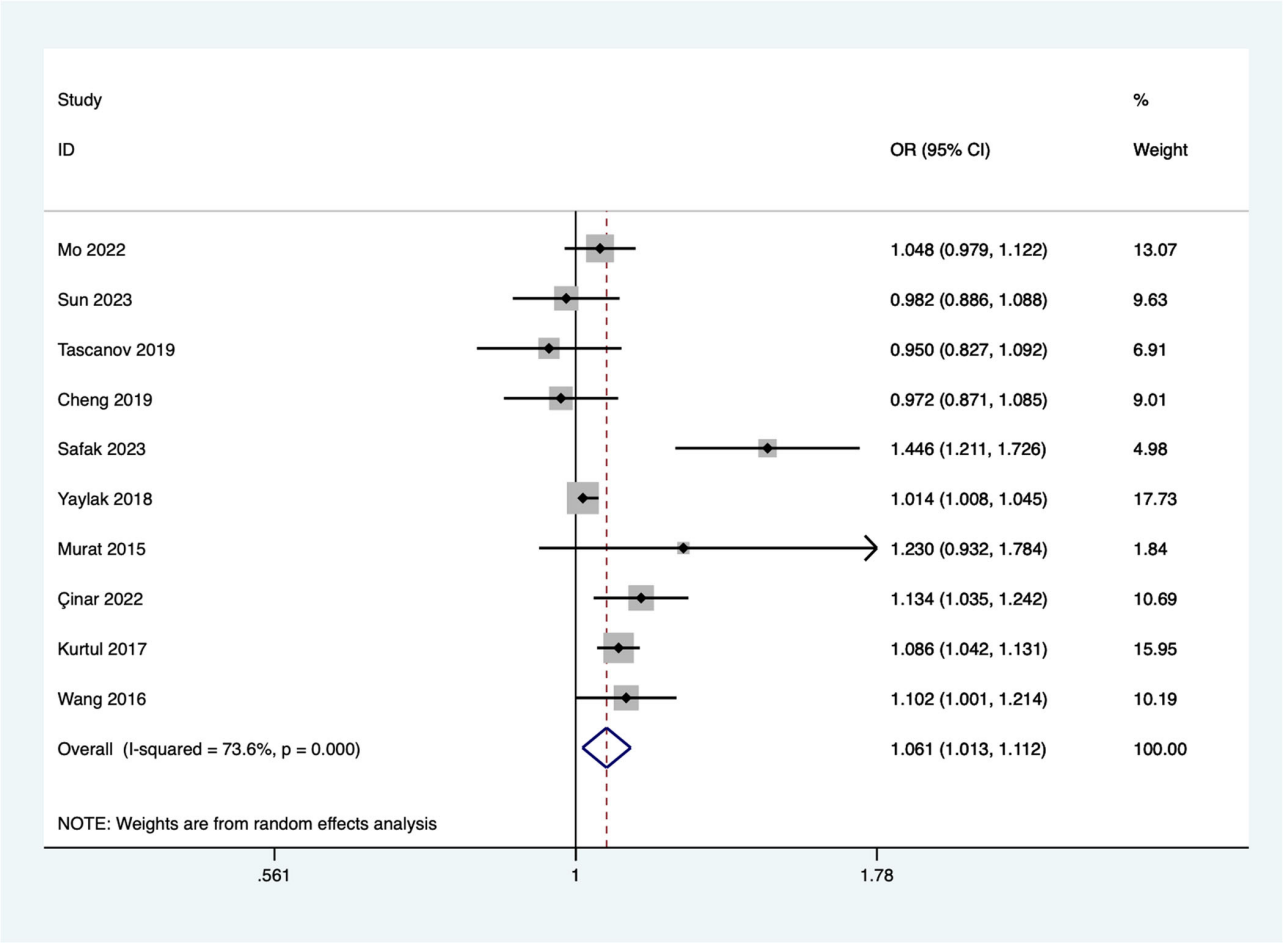
Association of white blood cell count and the risk of no‐reflow. CI, confidence interval; OR, odds ratio.

Ten studies investigated the relationship between WBC count and the risk of no‐reflow. Due to significant heterogeneity (*I* ^2^ = 73.6%, *p* = .000), a random‐effects model was employed for the meta‐analysis. The combined results revealed a significant association between an elevated WBC count and an increased risk of no‐reflow (OR = 1.061, 95% CI: 1.013–1.112, *p* = .012; see Figure 1).

##### Neutrophil count

**Figure 2:**
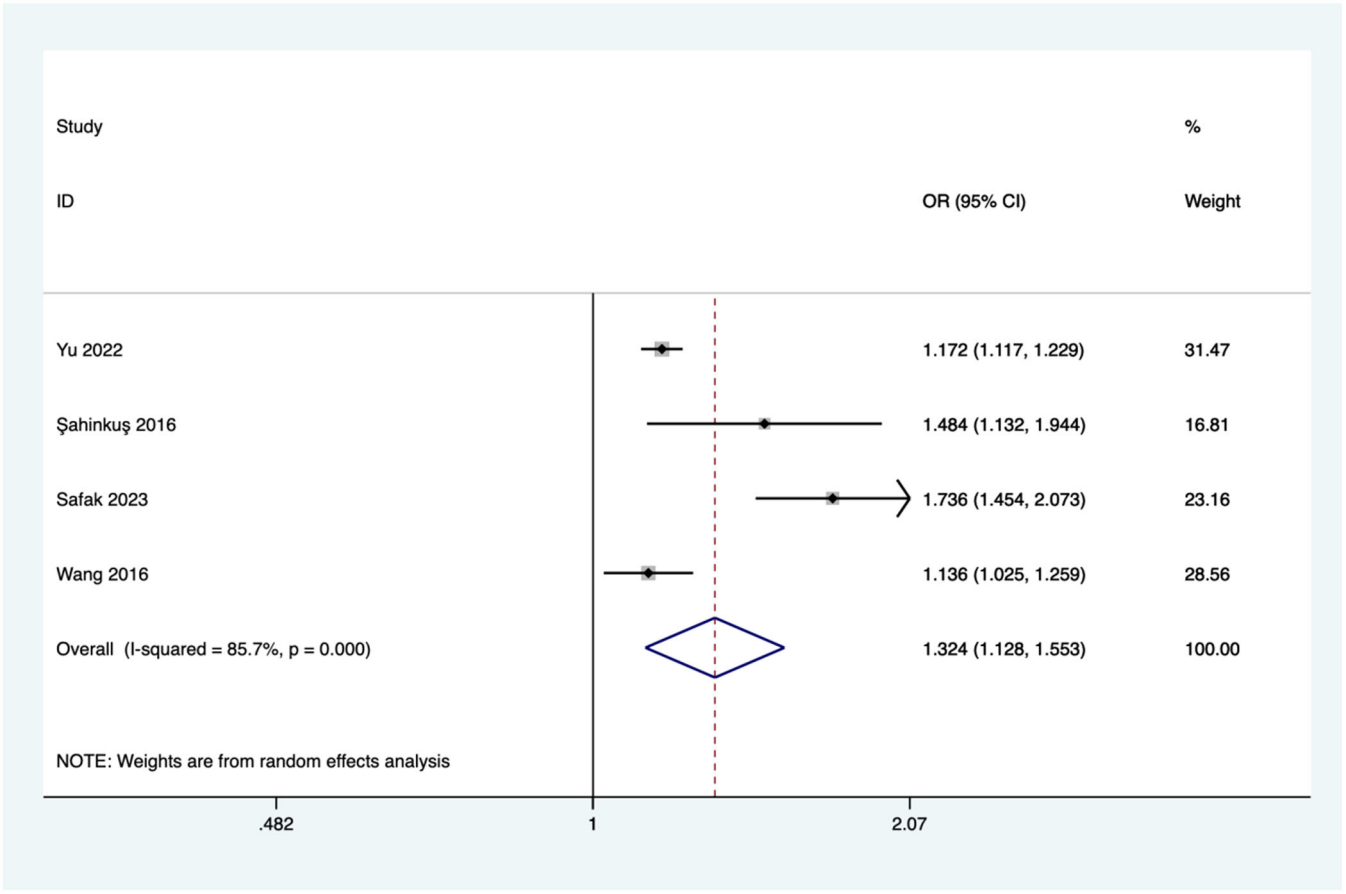
Association of white blood cell count and the risk of no‐reflow. CI, confidence interval; OR, odds ratio.

Four studies investigated the relationship between neutrophil count and the risk of no‐reflow. Due to significant heterogeneity (*I* ^2^ = 85.7%, *p* = .000), a random‐effects model was employed for the meta‐analysis. The combined results revealed a significant association between an elevated neutrophil count and an increased risk of no‐reflow (OR = 1.324, 95% CI: 1.128–1.553, *p* = .001; see Figure 2).

##### Lymphocyte count

**Figure 3:**
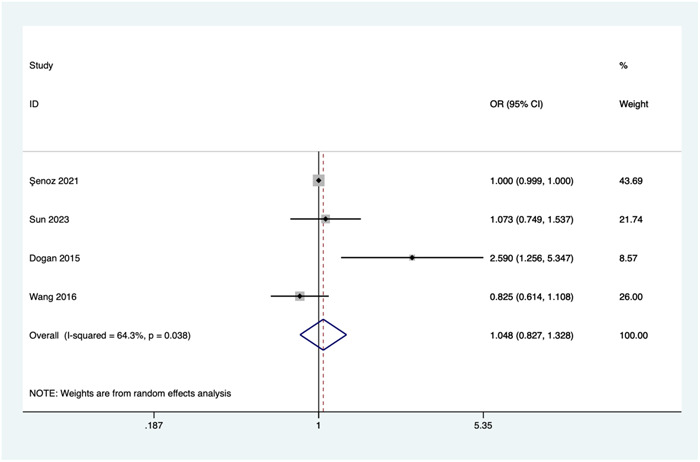
Association of lymphocyte count and the risk of no‐reflow. CI, confidence interval; OR, odds ratio.

Four studies investigated the relationship between lymphocyte count and the risk of no‐reflow. Due to significant heterogeneity (*I* ^2^ = 64.3%, *p* = .038), a random‐effects model was employed for the meta‐analysis. Pooled results show that there was no significant association between lymphocyte count and the risk of no‐reflow (OR = 1.048, 95%CI: 0.827–1.328, *p* = .698; see Figure 3).

##### Neutrophil‐to‐lymphocyte ratio (NLR)

**Figure 4:**
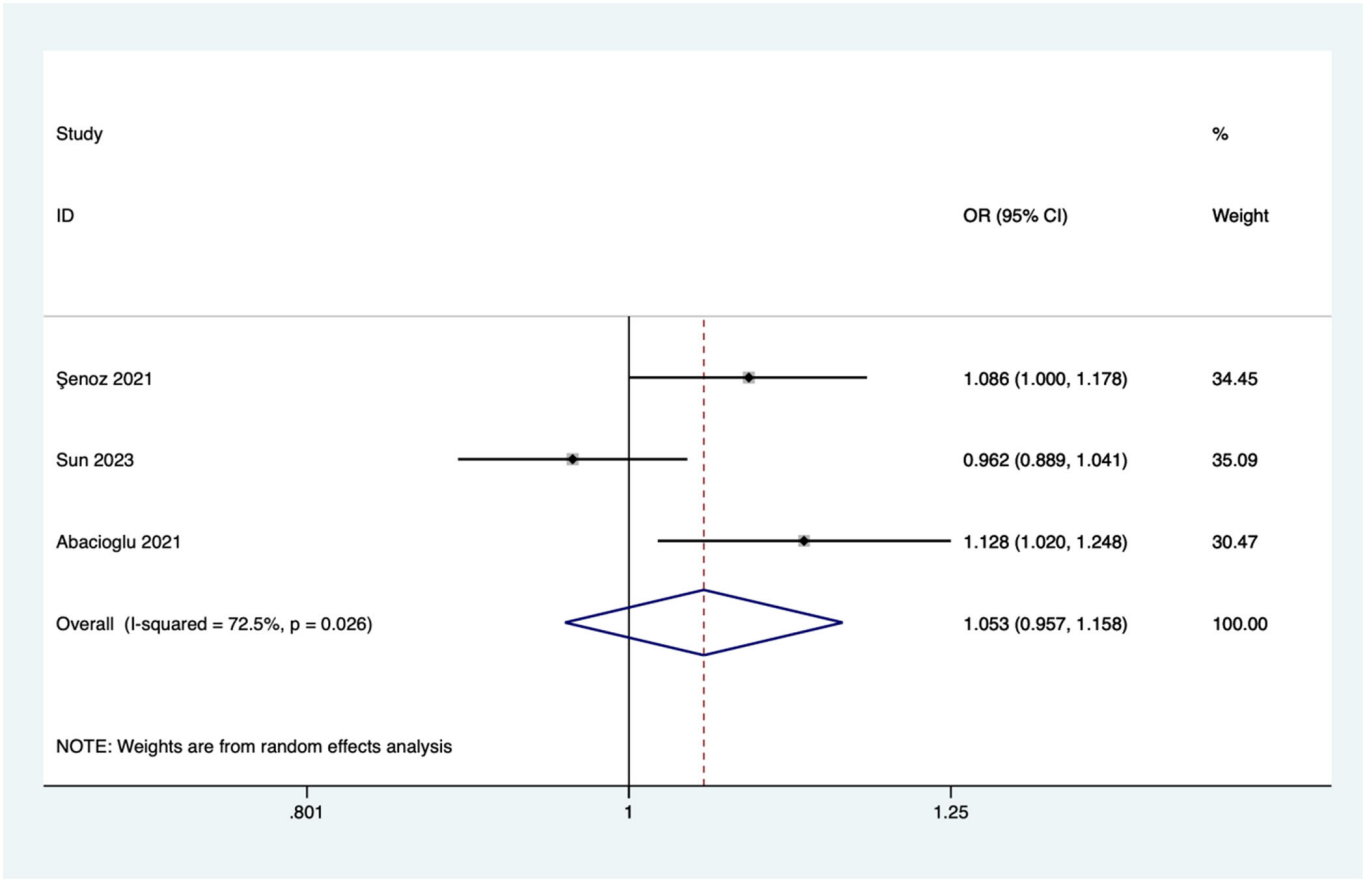
Association of neutrophil‐to‐lymphocyte ratio and the risk of no‐reflow. CI, confidence interval; OR, odds ratio.

Three studies investigated the relationship between NLR and the risk of no‐reflow. Due to significant heterogeneity (*I* ^2^ = 72.5%, *p* = .026), a random‐effects model was employed for the meta‐analysis. Pooled results show that there was no significant association between NLR and the risk of no‐reflow (OR = 1.053, 95% CI: 0.957–1.158, *p* = .291; see Figure 4).

#### 3.3.2 Platelet (PLT)

Nine studies investigated the relationship between PLT and the risk of no‐reflow. Due to significant heterogeneity (*I* ^2^ = 70.0%, *p* = .001), a random‐effects model was employed for the meta‐analysis. The combined results revealed a significant association between an elevated PLT and an increased risk of no‐reflow (OR = 1.002, 95% CI: 1.000–1.005, *p* = .038; see Figure S2).

#### 3.3.3 Blood glucose

Six studies investigated the relationship between blood glucose and the risk of no‐reflow. Due to significant heterogeneity (*I* ^2^ = 71.4%, *p* = .004), a random‐effects model was employed for the meta‐analysis. The combined results revealed a significant association between an elevated plasma glucose and an increased risk of no‐reflow (OR = 1.005, 95% CI: 1.002–1.009, *p* = .004; see Figure S3).

#### 3.3.4 Hemoglobin

Twelve studies investigated the relationship between hemoglobin and the risk of no‐reflow. Due to significant heterogeneity (*I* ^2^ = 97.5%, *p* = .000), a random‐effects model was employed for the meta‐analysis. The combined results revealed a significant association between an elevated hemoglobin and a decreased risk of no‐reflow (OR = 0.885, 95% CI: 1.002–1.009, *p* = .000; see Figure S4).

#### 3.3.5 Renal function index

##### Estimated GFR (eGFR)

Four studies explored the association between eGFR and the risk of no‐reflow. As there was no significant heterogeneity (*I* ^2^ = 45.1%, *p* = .141), a fixed‐effects model was used for the meta‐analysis. The combined results indicated no significant association between eGFR and the risk of no‐reflow (OR = 0.994, 95% CI: 0.986–1.001, *p* = .108; see Figure S5).

##### Creatinine

Five studies investigated the relationship between creatinine and the risk of no‐reflow. Due to significant heterogeneity (*I* ^2^ = 88.4%, *p* = .000), a random‐effects model was employed for the meta‐analysis. The combined results revealed a significant association between an elevated creatinine and an increased risk of no‐reflow (OR = 1.290, 95%CI: 1.070–1.555, *p* = .008; see Figure S6).

#### 3.3.6 Lipid index

##### Total cholesterol (TC)

Four studies investigated the relationship between TC and the risk of no‐reflow. As there was no significant heterogeneity (*I* ^2^ = 0.0%, *p* = .958), a fixed‐effects model was used for the meta‐analysis. The combined results revealed a significant association between an elevated TC and an increased risk of no‐reflow (OR = 1.022, 95%CI: 1.012–1.032, *p* = .000; see Figure S7).

##### TG

Ten studies explored the association between TG and the risk of no‐reflow. Due to significant heterogeneity (*I* ^2^ = 45.0%, *p* = .060), a random‐effects model was employed for the meta‐analysis. The combined results indicated no significant association between TG and the risk of no‐reflow (OR = 1.000, 95% CI: 0.997–1.004, *p* = .844; see Figure S8).

#### 3.3.7 Coagulation index

##### d‐dimer

Four studies investigated the relationship between d‐dimer and the risk of no‐reflow. As there was no significant heterogeneity (*I* ^2^ = 40.4%, *p* = .169), a fixed‐effects model was used for the meta‐analysis. The combined results revealed a significant association between an elevated d‐dimer and an increased risk of no‐reflow (OR = 1.002, 95% CI: 1.001–1.004, *p* = .008; see Figure S9).

##### Fibrinogen

Three studies investigated the relationship between fibrinogen and the risk of no‐reflow. As there was no significant heterogeneity (*I* ^2^ = 0.0%, *p* = .953), a fixed‐effects model was used for the meta‐analysis. The combined results revealed a significant association between an elevated fibrinogen and an increased risk of no‐reflow (OR = 1.010, 95% CI: 1.005–1.015, *p* = .008; see Figure S10).

#### 3.3.8 hsCRP

Five studies explored the association between hsCRP and the risk of no‐reflow. Due to significant heterogeneity (*I* ^2^ = 88.5%, *p* = .000), a random‐effects model was employed for the meta‐analysis. The combined results indicated no significant association between hsCRP and the risk of no‐reflow (OR = 1.052, 95% CI: 0.990–1.118, *p* = .105; see Figure S11).

### 3.4 Sensitivity analysis

By doing a meta‐analysis, we found that all the meta‐analyses did not have much effect on the results of the meta‐analysis, indicating that the results of the meta‐analysis were stable and reliable.

### 3.5 Publication bias

The funnel plot depicted in this study is presented below, revealing a predominantly symmetrical distribution. The obtained *p* value from Egger's test was .139, suggesting the absence of significant publication bias in this study (Figure S12).

## 4 CONCLUSION

In conclusion, our comprehensive analysis highlights the predictive potential of various parameters in assessing the risk of no‐reflow among STEMI patients undergoing PCI. Specifically, WBC count, neutrophil count, PLT, blood glucose, hemoglobin, creatinine, TC, d‐dimer, and fibrinogen emerged as significant predictors. These findings present valuable insights for clinicians, suggesting that incorporating these biomarkers into risk assessment protocols can enhance the identification of individuals at higher risk of experiencing no‐reflow post‐PCI in STEMI cases. This refined risk prediction may guide clinical decision‐making, allowing for more targeted and effective preventive measures to mitigate the occurrence of no‐reflow in this patient population.

## 5 DISCUSSION

The pathogenesis of no‐reflow and its associated risk factors are not fully understood. However, existing literature suggests several mechanisms that may contribute to this phenomenon, including (1) pre‐existing microvascular dysfunction; (2) distal microthromboembolization caused by elevated platelet activity and a significant thrombus burden; (3) ischemic injury; (4) reperfusion injury; (5) swelling of myocardial cells, resulting in the compression of microvascular vessels; and (6) individual susceptibility.^20^, ^48^, ^49^ While some studies have reported potential risk factors including advanced age, male, family history of coronary artery disease, smoking, diabetes mellitus, hypertension, and delayed reperfusion, these reports have been accompanied by inconsistencies. Furthermore, the relationship between laboratory parameters and the no‐reflow phenomenon remains uncertain. This study, for the first time, conducted a meta‐analysis to investigate the predictive role of laboratory parameters in the occurrence of the no‐reflow phenomenon, with the aim of offering guidance for clinical treatment.

Initially, the combined results indicated a noteworthy association between an elevated WBC count and an increased risk of no‐reflow (OR = 1.061). Particularly significant was the observation that an increased neutrophil count, a specific type of leukocyte, demonstrated a stronger association with an increased risk of no‐reflow (OR = 1.324 > 1.061). These findings suggest that the connection between WBC and the risk of no‐reflow is predominantly influenced by neutrophils. The findings confirm the involvement of microvascular inflammation in the pathogenesis of no‐reflow. This discovery provides concrete guidance for clinicians, enabling them to more accurately assess patients' risk of no‐reflow and implement targeted interventions. By incorporating neutrophil count into risk assessment and diagnostic criteria, clinicians can identify patients more precisely and offer personalized and precise treatment plans. This understanding also underscores the importance of inflammation in treatment strategies, opening new directions for future research and therapies. Overall, these findings offer substantial clinical guidance for improving the management of cardiovascular disease patients. Interestingly, a study conducted by Gullotta et al. in vivo demonstrated that in an experimental stroke, older mice exhibited greater neutrophil blockage in the microcirculation of the ischemic brain compared to younger mice, leading to more severe no‐reflow and poorer outcomes.^50^ This outcome provides further support for the objectivity of our analysis. Additionally, the combined results indicated a significant association between an elevated platelet count (PLT) and an increased risk of no‐reflow (OR = 1.002). However, the OR value was only 1.002, suggesting that platelet changes may not be a crucial factor in the occurrence of no‐reflow.

In addition to blood indices, we also examined the association of blood glucose and hemoglobin with the risk of no‐reflow. The aggregated results revealed a significant association between elevated blood glucose levels and an increased risk of no‐reflow, with an OR value of 1.005. Conversely, elevated hemoglobin levels were significantly associated with a lower risk of no‐reflow. This suggests that hemoglobin acts as a protective factor against the no‐reflow phenomenon, and the occurrence of no‐reflow can potentially be prevented by supplementing hemoglobin in clinical treatment. It is noteworthy that hemoglobin‐based oxygen carriers, such as Stroma‐free hemoglobin nanoparticles, have demonstrated neuroprotective effects in ischemia‐reperfusion injury.^51^

Moreover, elevated creatinine levels were identified as being associated with an increased risk of no‐reflow. This implies that individuals with STEMI and impaired renal function should exercise particular caution to prevent no‐reflow after PCI. Additionally, increased levels of TC, d‐dimer, and fibrinogen were also significantly associated with an elevated risk of no‐reflow. This suggests that lipid accumulation and sluggish blood flow may contribute to the occurrence of no‐reflow.

This meta‐analysis has several limitations. Firstly, heterogeneity was observed in the studies of some indicators, and while sensitivity analysis revealed no studies significantly influencing the results, the source of heterogeneity may be attributed to variations in the correction of results. This study extracted OR values from regression analysis sources for summary, but only a subset of studies corrected their results. Secondly, the included literature was obtained through electronic searches and comprised solely of published studies. Unpublished literature was not taken into consideration. Third, other hemogram parameters‐based markers (e.g., PLR, SII, PIV) did not meet the criteria for inclusion in the meta‐analysis for the studies included in this research. Therefore, analysis of these markers was not feasible in the current study. Future investigations will necessitate the inclusion of additional studies to facilitate the analysis of hemogram parameters‐based markers beyond NLR.

## AUTHOR CONTRIBUTIONS

LinLi Wang and Qiujun Zhou performed the research. Qiujun Zhou and ShuWei Huang designed the research study. LiPing Dou and Qiujun Zhou participated in data collection. Dongming Lin analyzed the data. Dongming Lin and LinLi Wang wrote and revised the paper. All authors have read and approved the final manuscript.

## CONFLICT OF INTEREST STATEMENT

The authors declare no conflict of interest.

## Supporting information

Supporting information.

Supporting information.

Supporting information.

Supporting information.

Supporting information.

Supporting information.

Supporting information.

Supporting information.

Supporting information.

Supporting information.

Supporting information.

Supporting information.

Supporting information.

Supporting information.

Supporting information.

Supporting information.

Supporting information.

Supporting information.

Supporting information.

Supporting information.

Supporting information.

Supporting information.

Supporting information.

Supporting information.

Supporting information.

## Data Availability

The data that support the findings of this study are available from the corresponding author upon reasonable request.

## References

[1] Grüntzig AR , Senning Å , Siegenthaler WE . Nonoperative dilatation of coronary‐artery stenosis: percutaneous transluminal coronary angioplasty. N Engl J Med. 1979;301:61‐68. 449946 10.1056/NEJM197907123010201

[2] Steg PG . Primary percutaneous coronary intervention in acute myocardial infarction: time, time, and time! Heart. 2005;91:993‐994. 16020578 10.1136/hrt.2004.050625 PMC1769058

[3] Mohar DS , Seto AH , Kern MJ . Primary percutaneous coronary intervention in patients with ST‐segment‐elevation myocardial infarction and concurrent active gastrointestinal bleeding. Circ Cardiovasc Interv. 2015;8(10):e003058. 26450353 10.1161/CIRCINTERVENTIONS.115.003058

[4] Gupta S , Gupta MM . No reflow phenomenon in percutaneous coronary interventions in ST‐segment elevation myocardial infarction. Indian Heart J. 2016;68:539‐551. 27543480 10.1016/j.ihj.2016.04.006 PMC4990737

[5] Hagen TP , Häkkinen U , Belicza E , Fatore G , Goude F . Acute myocardial infarction, use of percutaneous coronary intervention, and mortality: a comparative effectiveness analysis covering seven european countries. Health Econ. 2015;24(suppl 2):88‐101. 26633870 10.1002/hec.3263

[6] Rott D . Advantage of percutaneous coronary intervention over medical therapy in angina relief and the placebo effect. JACC. 2005;45:327‐328; 10.1016/j.jacc.2004.10.026 15653043

[7] Goff SL , Mazor KM , Ting HH , Kleppel R , Rothberg MB . How cardiologists present the benefits of percutaneous coronary interventions to patients with stable angina: a qualitative analysis. JAMA Intern Med. 2014;174:1614‐1621. 25156523 10.1001/jamainternmed.2014.3328 PMC4553927

[8] Singh AK . Percutaneous coronary intervention vs coronary artery bypass grafting in the management of chronic stable angina: a critical appraisal. J Cardiovasc Dis Res. 2010;1:54‐58. 20877686 10.1016/s0975-3583(10)12003-8 PMC2945205

[9] Stouffer GA , Lenihan D , Lerakis S , et al. Incidence and management of “no‐reflow” following percutaneous coronary interventions. Am J Med Sci. 2005;329:78‐85. 15711424 10.1097/00000441-200502000-00005

[10] Choo EH , Kim PJ , Chang K , et al. The impact of no‐reflow phenomena after primary percutaneous coronary intervention: a time‐dependent analysis of mortality. Coron Artery Dis. 2014;25:392‐398. 24625688 10.1097/MCA.0000000000000108

[11] Jaffe R , Charron T , Puley G , Dick A , Strauss BH . Microvascular obstruction and the no‐reflow phenomenon after percutaneous coronary intervention. Circulation. 2008;117:3152‐3156. 18559715 10.1161/CIRCULATIONAHA.107.742312

[12] Wang L , Cheng Z , Gu Y , Peng D . Short‐term effects of verapamil and diltiazem in the treatment of no reflow phenomenon: a meta‐analysis of randomized controlled trials. BioMed Res Int. 2015;2015:382086. 26504804 10.1155/2015/382086 PMC4609355

[13] Abu Arab T , Rafik R , El Etriby A . Efficacy and safety of local intracoronary drug delivery in treatment of no‐reflow phenomenon: a pilot study. J Interv Cardiol. 2016;29:496‐504. 27465353 10.1111/joic.12318

[14] Ramjane K , Han L , Jin C . The diagnosis and treatment of the no‐reflow phenomenon in patients with myocardial infarction undergoing percutaneous coronary intervention. Exp Clin Cardiol. 2008;13:121‐128. 19343126 PMC2586408

[15] Niccoli G , Rigattieri S , De Vita MR , et al. Open‐label, randomized, placebo‐controlled evaluation of intracoronary adenosine or nitroprusside after thrombus aspiration during primary percutaneous coronary intervention for the prevention of microvascular obstruction in acute myocardial infarction: the REOPEN‐AMI study (Intracoronary Nitroprusside Versus Adenosine in Acute Myocardial Infarction). JACC Cardiovasc Interv. 2013;6:580‐589. 23683738 10.1016/j.jcin.2013.02.009

[16] Prati F , Romagnoli E , Limbruno U , et al. Randomized evaluation of intralesion versus intracoronary abciximab and aspiration thrombectomy in patients with ST‐elevation myocardial infarction: the COCTAIL II trial. Am Heart J. 2015;170:1116‐1123. 26678633 10.1016/j.ahj.2015.08.020

[17] Sakakura K , Funayama H , Taniguchi Y , et al. The incidence of slow flow after rotational atherectomy of calcified coronary arteries: A randomized study of low speed versus high speed. Catheter Cardiovasc Interv. 2017;89:832‐840. 27453426 10.1002/ccd.26698

[18] Resnic FS , Wainstein M , Lee MKY , et al. No‐reflow is an independent predictor of death and myocardial infarction after percutaneous coronary intervention. Am Heart J. 2003;145:42‐46. 12514653 10.1067/mhj.2003.36

[19] Harrison RW , Aggarwal A , Ou F , et al. Incidence and outcomes of no‐reflow phenomenon during percutaneous coronary intervention among patients with acute myocardial infarction. Am J Cardiol. 2013;111:178‐184. 23111142 10.1016/j.amjcard.2012.09.015

[20] Wong DTL , Puri R , Richardson JD , Worthley MI , Worthley SG . Myocardial ‘no‐reflow’—diagnosis, pathophysiology and treatment. Int J Cardiol. 2013;167:1798‐1806. 23357047 10.1016/j.ijcard.2012.12.049

[21] Cook DA , Reed DA . Appraising the quality of medical education research methods: the medical education research study quality instrument and the Newcastle‐Ottawa Scale‐Education. Acad Med. 2015;90:1067‐1076. 26107881 10.1097/ACM.0000000000000786

[22] Rethlefsen ML , Kirtley S , Waffenschmidt S , et al. PRISMA‐S: an extension to the PRISMA statement for reporting literature searches in systematic reviews. Syst Rev. 2021;10:39. 33499930 10.1186/s13643-020-01542-z PMC7839230

[23] Xu J , Geng J , Zhang Q , Fan Y , Qi Z , Xia T . Association of three micro‐RNA gene polymorphisms with the risk of cervical cancer: a meta‐analysis and systematic review. World J Surg Oncol. 2021;19:346. 34911543 10.1186/s12957-021-02463-4 PMC8675500

[24] Mo DG , Wang CS , Liu JH , Li T . The predictive value of eosinophil levels on no‐reflow in patients with STEMI following PCI: a retrospective cohort study. Sci Rep. 2022;12:17862. 36284176 10.1038/s41598-022-22988-2 PMC9596413

[25] Şimşek B , Çınar T , Ozan V , et al. The association of acute­‐to­‐chronic glycemic ratio with no‐reflow in patients with ST­‐segment elevation myocardial infarction undergoing primary percutaneous coronary intervention. Kardiol Pol. 2021;79:170‐178. 33394580 10.33963/KP.15736

[26] Li Q , Xie E , Tu Y , et al. Association between kaolin‐induced maximum amplitude and slow‐flow/no‐reflow in ST elevation myocardial infarction patients treated with primary percutaneous coronary intervention. Int J Cardiol. 2022;369:13‐18. 35970443 10.1016/j.ijcard.2022.08.025

[27] Yu Y , Wu Y , Wu X , Wang J , Wang C . Risk factors for no‐reflow in patients with ST‐elevation myocardial infarction who underwent percutaneous coronary intervention: a case‐control study. Cardiol Res Pract. 2022;2022:3482518. 35308062 10.1155/2022/3482518 PMC8930256

[28] Zhang Q , Hu M. , Ma S. , Niu T. et al. New R(2)‐CHA(2)DS(2)‐VASc score predicts no‐reflow phenomenon and long‐term prognosis in patients with ST‐segment elevation myocardial infarction after primary percutaneous coronary intervention. Front Cardiovasc Med. 2022;9:899739. 36312233 10.3389/fcvm.2022.899739 PMC9609412

[29] Liu Y , Ye T , Chen K , et al. A nomogram risk prediction model for no‐reflow after primary percutaneous coronary intervention based on rapidly accessible patient data among patients with ST‐segment elevation myocardial infarction and its relationship with prognosis. Front Cardiovasc Med. 2022;9:966299. 36003914 10.3389/fcvm.2022.966299 PMC9393359

[30] Sahinkus S , Cakar M , Yaylaci S , et al. Hematological markers of the no‐reflow phenomen on in‐patients undergoing primary percutaneous coronary intervention. Georgian Med News. 2016;(254):26‐32. 27348163

[31] Senoz O , Emren SV , Ersecgin A , Yapan Emren Z , Gul I . Platelet‐Lymphocyte ratio is a predictor for the development of no‐reflow phenomenon in patients with ST‐segment elevation myocardial infarction after thrombus aspiration. J Clin Lab Anal. 2021;35:e23795. 33945171 10.1002/jcla.23795 PMC8183944

[32] Sun Y , Ren J , Li L , Wang C , Yao H . RDW as a predictor for no‐reflow phenomenon in DM patients with ST‐segment elevation myocardial infarction undergoing primary percutaneous coronary intervention. J Clin Med. 2023;12:807. 36769459 10.3390/jcm12030807 PMC9917933

[33] Liu F , Huang R , Li Y , Zhao S , Gong Y , Xu Z . In‐Hospital peak glycemia in predicting no‐reflow phenomenon in diabetic patients with STEMI treated with primary percutaneous coronary intervention. J Diabetes Res. 2021;2021:6683937. 33506051 10.1155/2021/6683937 PMC7811415

[34] Tascanov MB , Tanriverdi Z , Gungoren F , et al. Association between the no‐reflow phenomenon and soluble CD40 ligand level in patients with acute ST‐segment elevation myocardial infarction. Medicina. 2019;55:376. 31311177 10.3390/medicina55070376 PMC6681218

[35] Dogan NB , Ozpelit E , Akdeniz S , Bilgin M , Baris N . Simple clinical risk score for no‐reflow prediction in patients undergoing primary percutaneous coronary intervention with acute STEMI. Pak J Med Sci. 2015;31:576‐581. 26150847 10.12669/pjms.313.7484 PMC4485274

[36] Cheng C , Liu XB , Bi SJ , Lu QH , Zhang J . Serum cystatin C levels relate to no‐reflow phenomenon in percutaneous coronary interventions in ST‐segment elevation myocardial infarction. PloS one. 2019;14:e0220654. 31369621 10.1371/journal.pone.0220654 PMC6675089

[37] Safak O , Yildirim T , Emren V , et al. Prognostic nutritional index as a predictor of no‐reflow occurrence in patients with ST‐segment elevation myocardial infarction who underwent primary percutaneous coronary intervention. Angiology. 2016;27(2):116‐121. 10.1177/00033197231193223 37553838

[38] Yaylak B , Altıntaş B , Özcan KS , et al. Relation of hemoglobin level to no‐reflow in patients with ST‐segment elevation myocardial infarction undergoing primary coronary intervention. Adv Interv Cardiol. 2018;14:383‐390. 10.5114/aic.2018.79868 PMC6309849 30603028

[39] Yildirim A , Kucukosmanoglu M , Koyunsever NY , Cekici Y , Belibagli MC , Kilic S . Relationship between blood viscosity and no‐reflow phenomenon in ST‐segment elevation myocardial infarction performed in primary percutaneous coronary interventions. Biomark Med. 2021;15:659‐667. 34039016 10.2217/bmm-2020-0772

[40] Dogdus M , Yenercag M , Ozyasar M , Yilmaz A , Can LH , Kultursay H . Serum endocan levels predict angiographic no‐reflow phenomenon in patients with ST‐segment elevation myocardial infarction undergoing primary coronary intervention. Angiology. 2021;72:221‐227. 32996338 10.1177/0003319720961954

[41] Murat SN , Kurtul A , Celik IE , et al. The association of serum procalcitonin level with the no‐reflow phenomenon after a primary percutaneous coronary intervention in patients with ST‐elevation myocardial infarction. Coron Artery Dis. 2016;27:116‐121. 26709984 10.1097/MCA.0000000000000329

[42] Çınar T , Şaylık F , Hayıroğlu MI , et al. The association of serum uric acid/albumin ratio with no‐reflow in patients with ST elevation myocardial infarction. Angiology. 2023;74:381‐386. 35726733 10.1177/00033197221110700

[43] Abacioglu OO , Yildirim A , Koyunsever NY , Kilic S . The ATRIA and modified‐ATRIA scores in evaluating the risk of no‐reflow in patients with STEMI undergoing primary percutaneous coronary intervention. Angiology. 2022;73:79‐84. 34180260 10.1177/00033197211026420

[44] Zhao Y , Yang J , Ji Y , et al. Usefulness of fibrinogen‐to‐albumin ratio to predict no‐reflow and short‐term prognosis in patients with ST‐segment elevation myocardial infarction undergoing primary percutaneous coronary intervention. Heart Vessels. 2019;34:1600‐1607. 30993442 10.1007/s00380-019-01399-w

[45] Kurtul A , Acikgoz SK . Usefulness of mean platelet volume‐to‐lymphocyte ratio for predicting angiographic no‐reflow and short‐term prognosis after primary percutaneous coronary intervention in patients with ST‐segment elevation myocardial infarction. Am J Cardiol. 2017;120:534‐541. 28633762 10.1016/j.amjcard.2017.05.020

[46] Kurtul A , Yarlioglues M , Murat SN , et al. Usefulness of the platelet‐to‐lymphocyte ratio in predicting angiographic reflow after primary percutaneous coronary intervention in patients with acute ST‐segment elevation myocardial infarction. Am J Cardiol. 2014;114:342‐347. 24948493 10.1016/j.amjcard.2014.04.045

[47] Wang Z , Ren L , Liu N , Lei L , Ye H , Peng J . Association of monocyte count on admission with angiographic no‐reflow after primary percutaneous coronary intervention in patients with ST‐segment elevation myocardial infarction. Kardiol Pol. 2016;74:1160‐1166. 27160177 10.5603/KP.a2016.0065

[48] Zhou H , He X , Zhuang S , et al. Clinical and procedural predictors of no‐reflow in patients with acute myocardial infarction after primary percutaneous coronary intervention. World J Emerg Med. 2014;5:96‐102. 25215156 10.5847/wjem.j.issn.1920-8642.2014.02.003 PMC4129879

[49] Bolayir HA , Gunes H , Kivrak T , et al. The role of SCUBE1 in the pathogenesis of no‐reflow phenomenon presenting with ST segment elevation myocardial infarction. Anatol J Cardiol. 2017;18:122‐127. 28554990 10.14744/AnatolJCardiol.2017.7705 PMC5731261

[50] Gullotta GS , De Feo D , Friebel E , et al. Age‐induced alterations of granulopoiesis generate atypical neutrophils that aggravate stroke pathology. Nat Immunol. 2023;24:925‐940. 37188941 10.1038/s41590-023-01505-1

[51] Tatezawa R , Abumiya T , Ito Y , et al. Neuroprotective effects of a hemoglobin‐based oxygen carrier (stroma‐free hemoglobin nanoparticle) on ischemia reperfusion injury. Brain Res. 1821;2023:148592. 10.1016/j.brainres.2023.148592 37748569

