# Supplementary material for: The predictive value of laboratory parameters for no‐reflow phenomenon in patients with ST‐elevation myocardial infarction following primary percutaneous coronary intervention: A meta‐analysis
Source: Clin Cardiol. 2024 Feb 23;47(2):e24238. doi: 10.1002/clc.24238 (PMC10891415; doi:10.1002/clc.24238)
Supplement: Supplementary file 1 — Supporting information. [file CLC-47-e24238-s016.docx]

**Legends**

Figure S1. Flow diagram for selection of studies.

Table 1. Baseline characteristics and quality assessment of the included studies

Figure 1. Association of WBC count and the risk of no-reflow

Figure 2. Association of WBC count and the risk of no-reflow

Figure 3. Association of lymphocyte count and the risk of no-reflow

Figure 4. Association of NLR and the risk of no-reflow

Figure S1. Flow diagram for selection of studies

Figure S2. Association of PLT and the risk of no-reflow

Figure S3. Association of blood glucose and the risk of no-reflow

Figure S4. Association of hemoglobin and the risk of no-reflow

Figure S5. Association of eGFR and the risk of no-reflow

Figure S6. Association of creatinine and the risk of no-reflow

Figure S7. Association of TC and the risk of no-reflow

Figure S8. Association of TG and the risk of no-reflow

Figure S9. Association of D-dimer and the risk of no-reflow

Figure S10. Association of fibrinogen and the risk of no-reflow

Figure S11. Association of hsCRP and the risk of no-reflow

Figure S12. Funnel plot for evaluating the publication bias of this meta-analysis
