# Supplementary figures and images for: The predictive value of laboratory parameters for no‐reflow phenomenon in patients with ST‐elevation myocardial infarction following primary percutaneous coronary intervention: A meta‐analysis
Source: Clin Cardiol. 2024 Feb 23;47(2):e24238. doi: 10.1002/clc.24238 (PMC10891415; doi:10.1002/clc.24238)

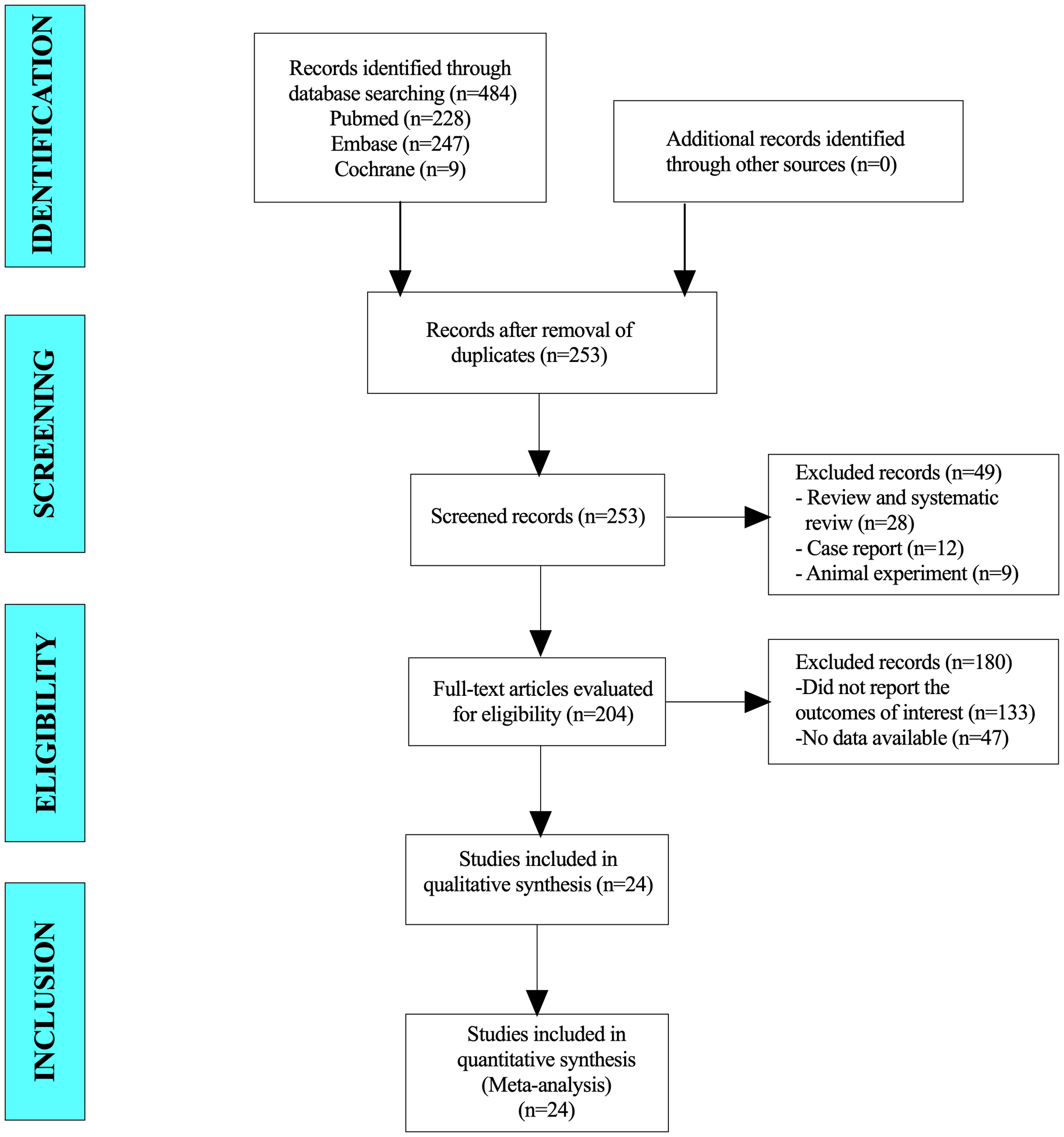

Supplement: Supplementary file 2 — Supporting information. [file CLC-47-e24238-s013.tiff]

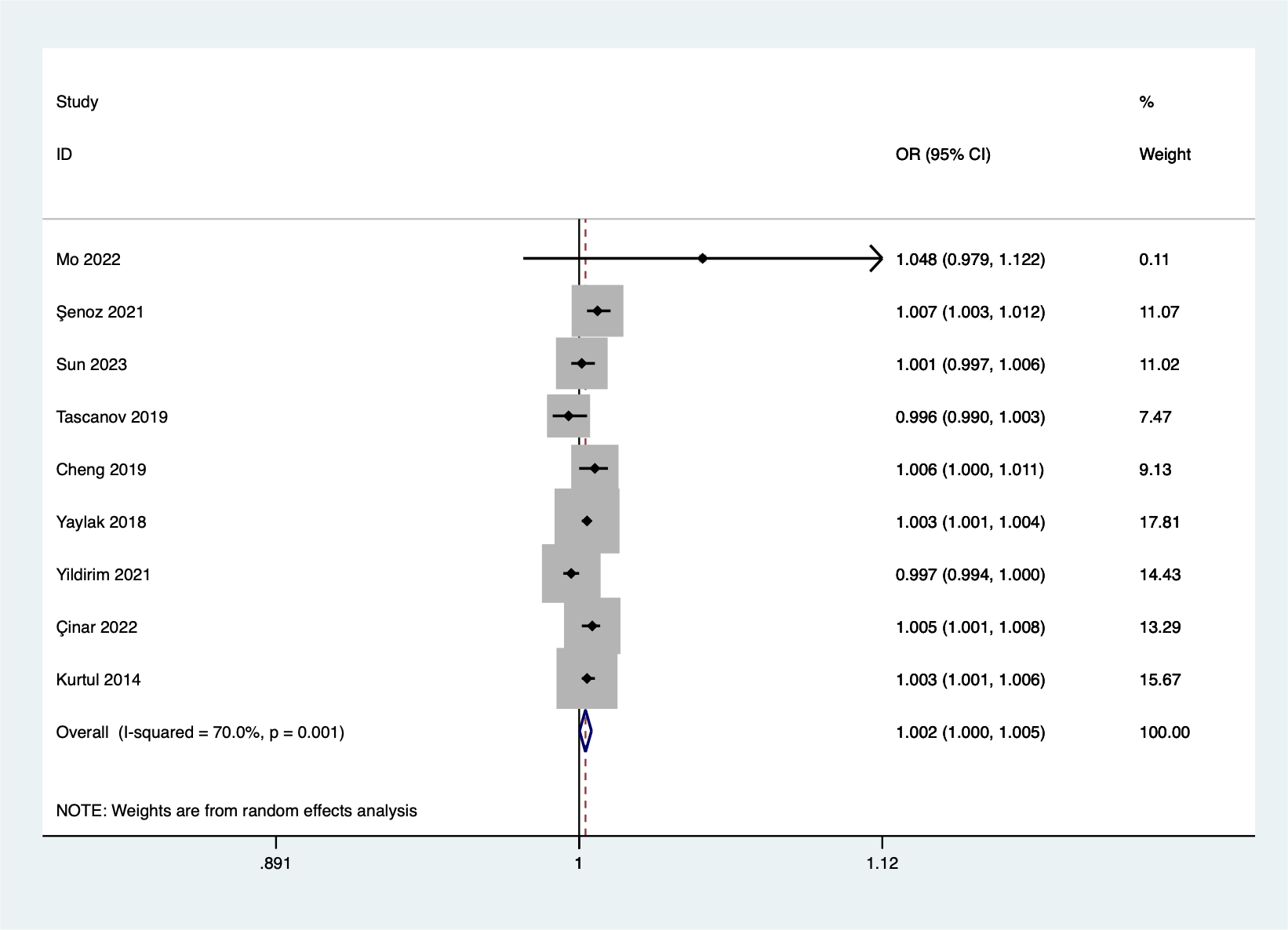

Supplement: Supplementary file 4 — Supporting information. [file CLC-47-e24238-s012.tif]

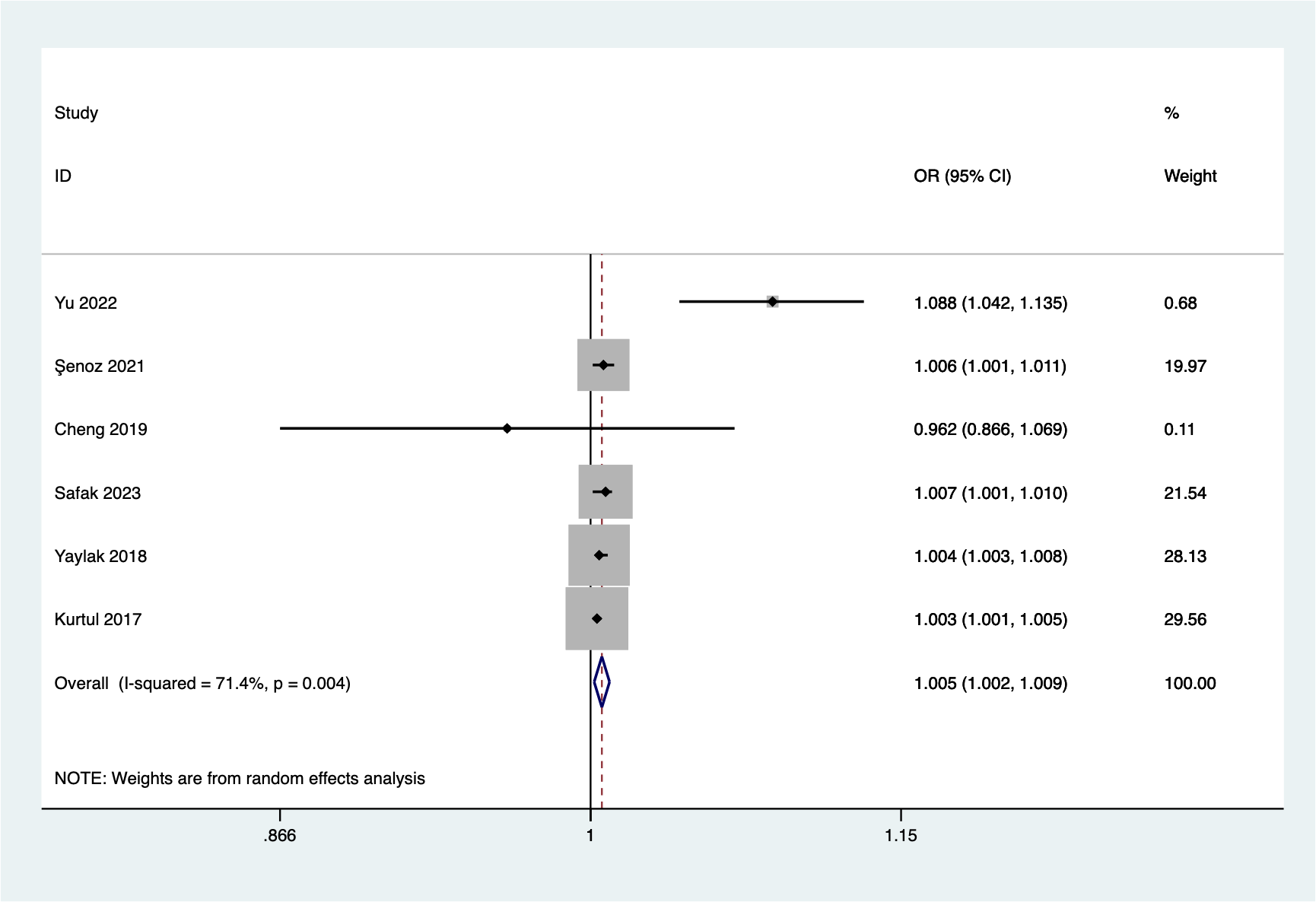

Supplement: Supplementary file 6 — Supporting information. [file CLC-47-e24238-s002.tif]

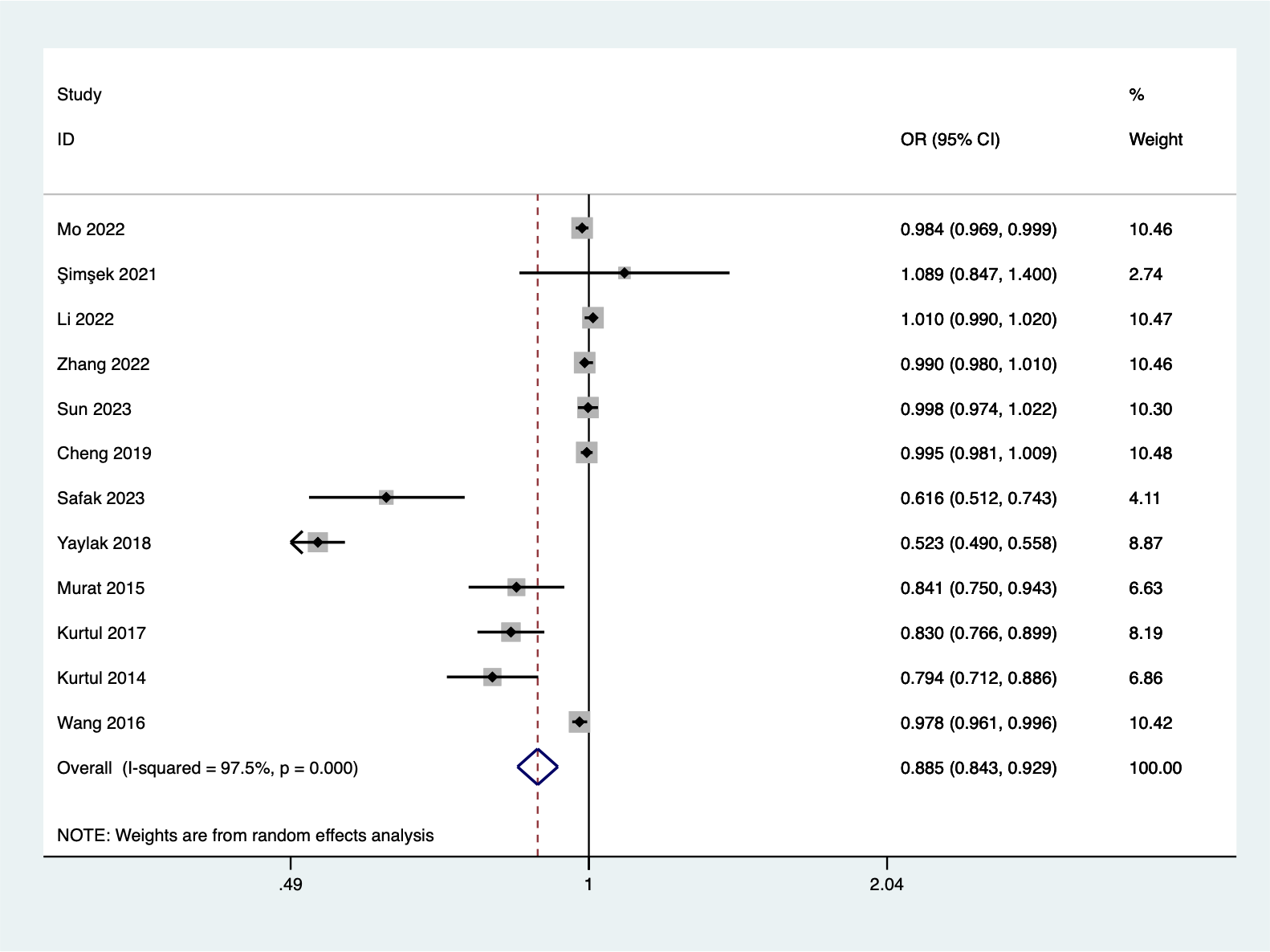

Supplement: Supplementary file 8 — Supporting information. [file CLC-47-e24238-s017.tif]

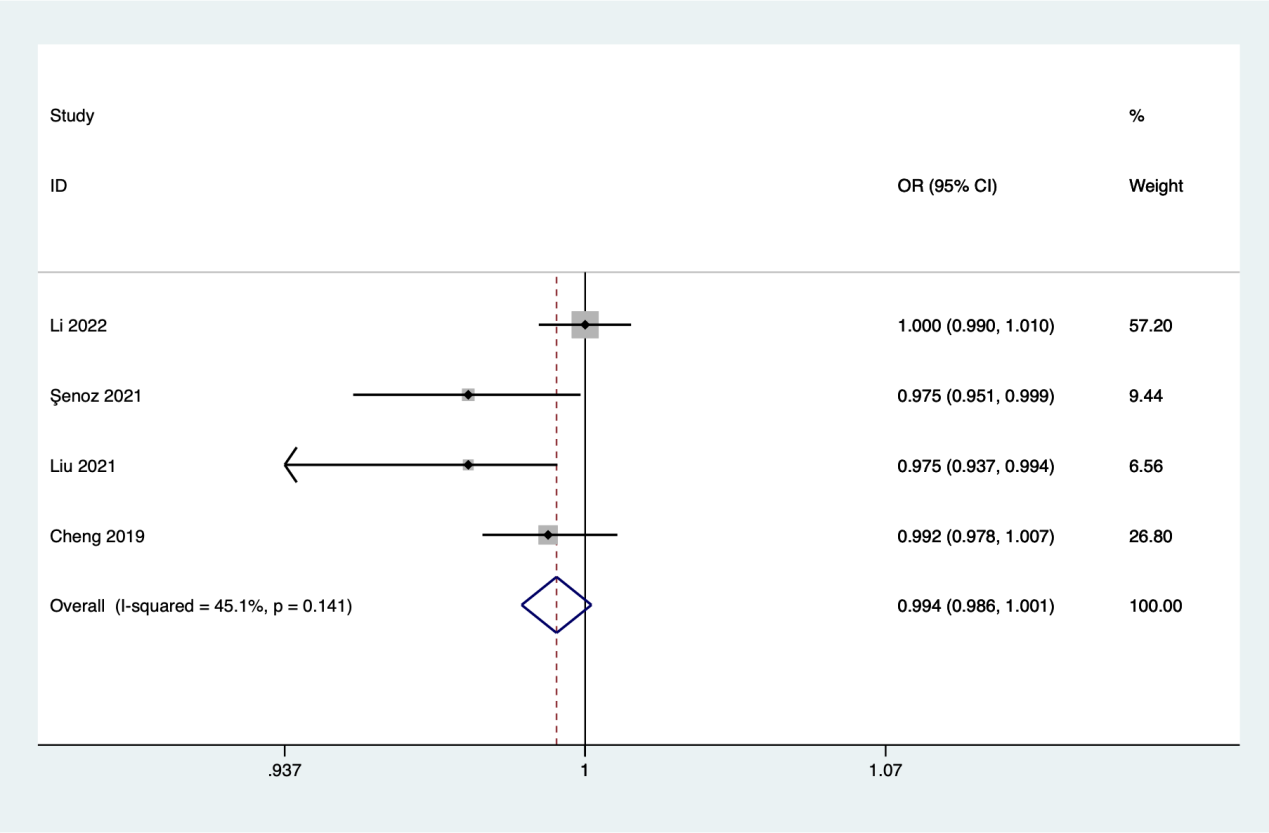

Supplement: Supplementary file 10 — Supporting information. [file CLC-47-e24238-s009.tif]

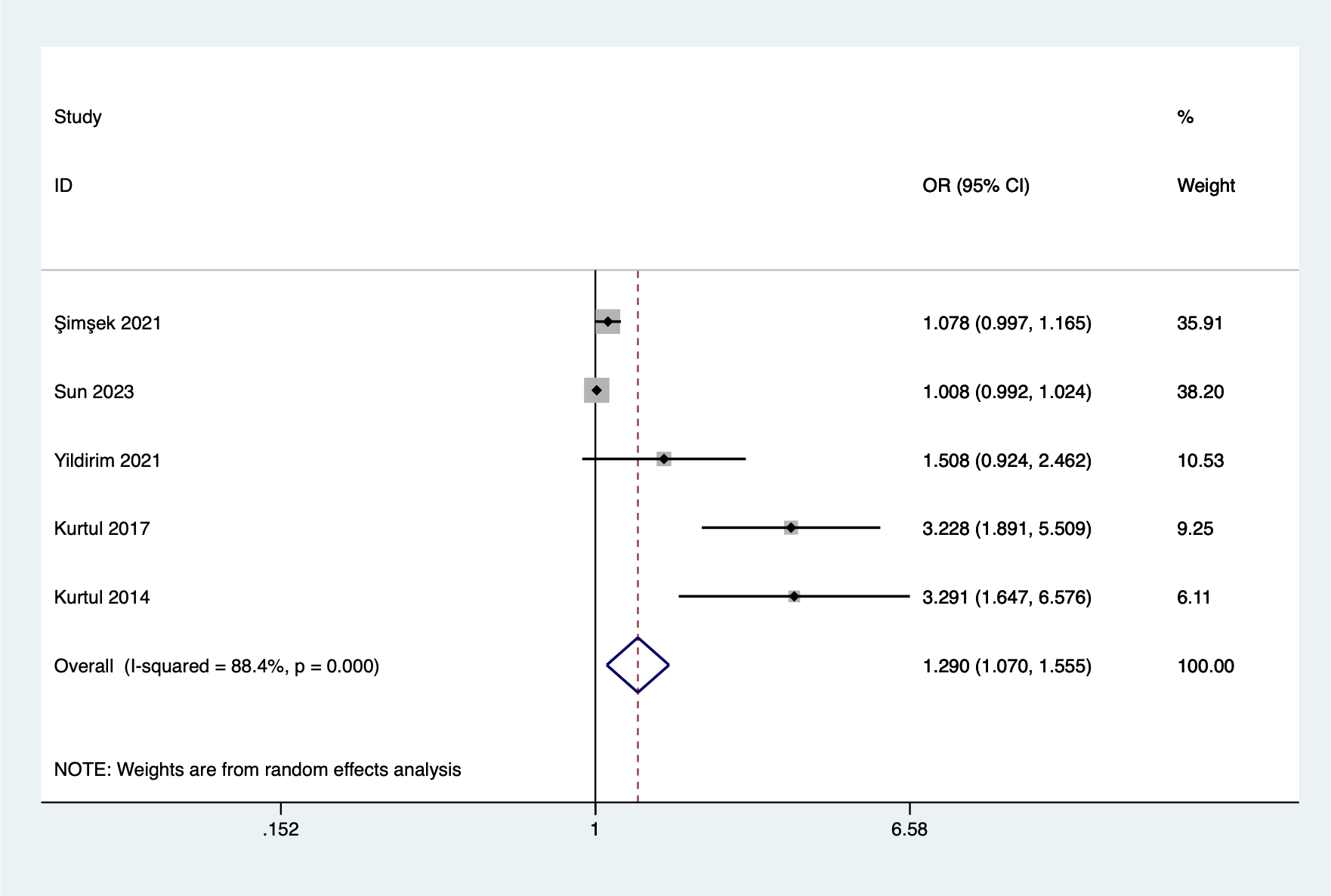

Supplement: Supplementary file 12 — Supporting information. [file CLC-47-e24238-s023.tif]

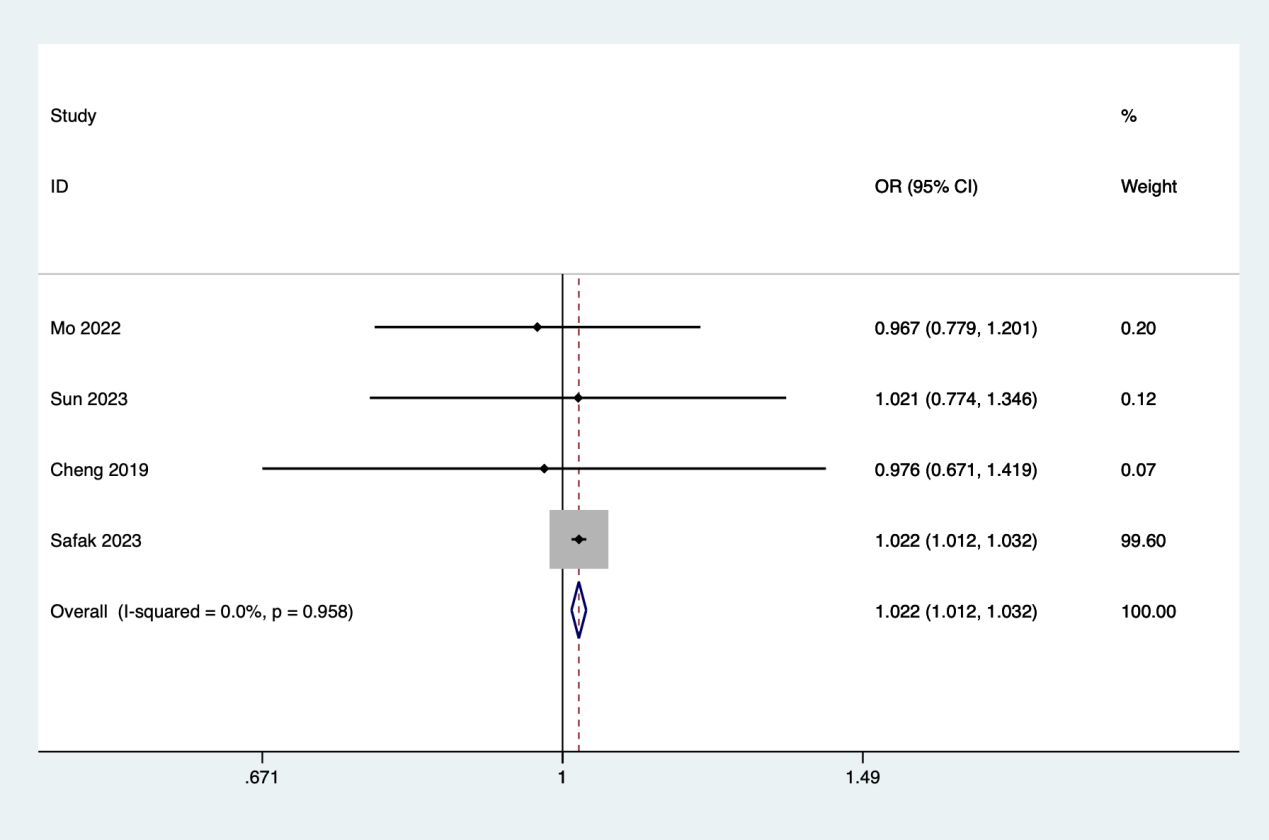

Supplement: Supplementary file 14 — Supporting information. [file CLC-47-e24238-s022.tif]

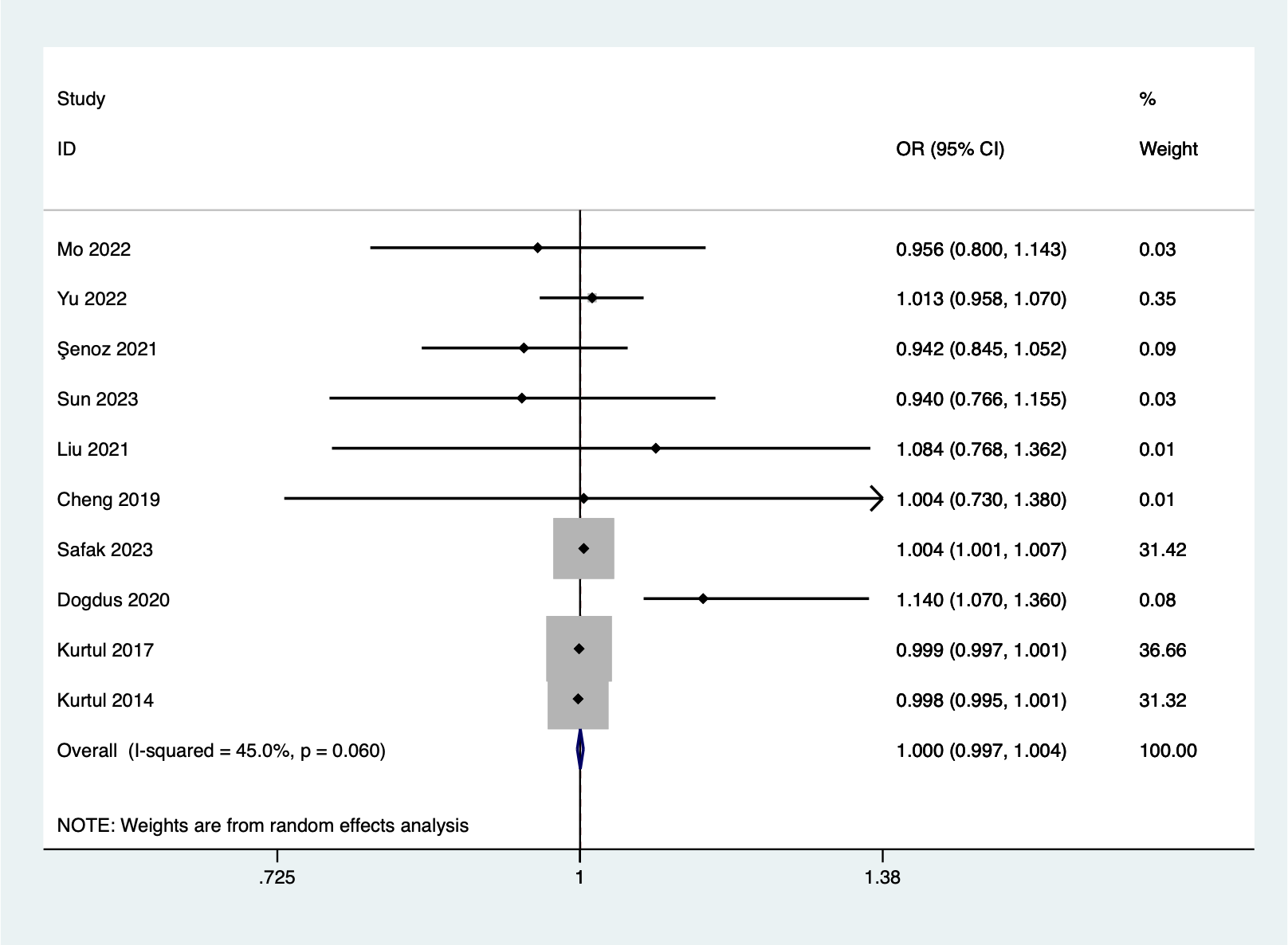

Supplement: Supplementary file 16 — Supporting information. [file CLC-47-e24238-s024.tif]

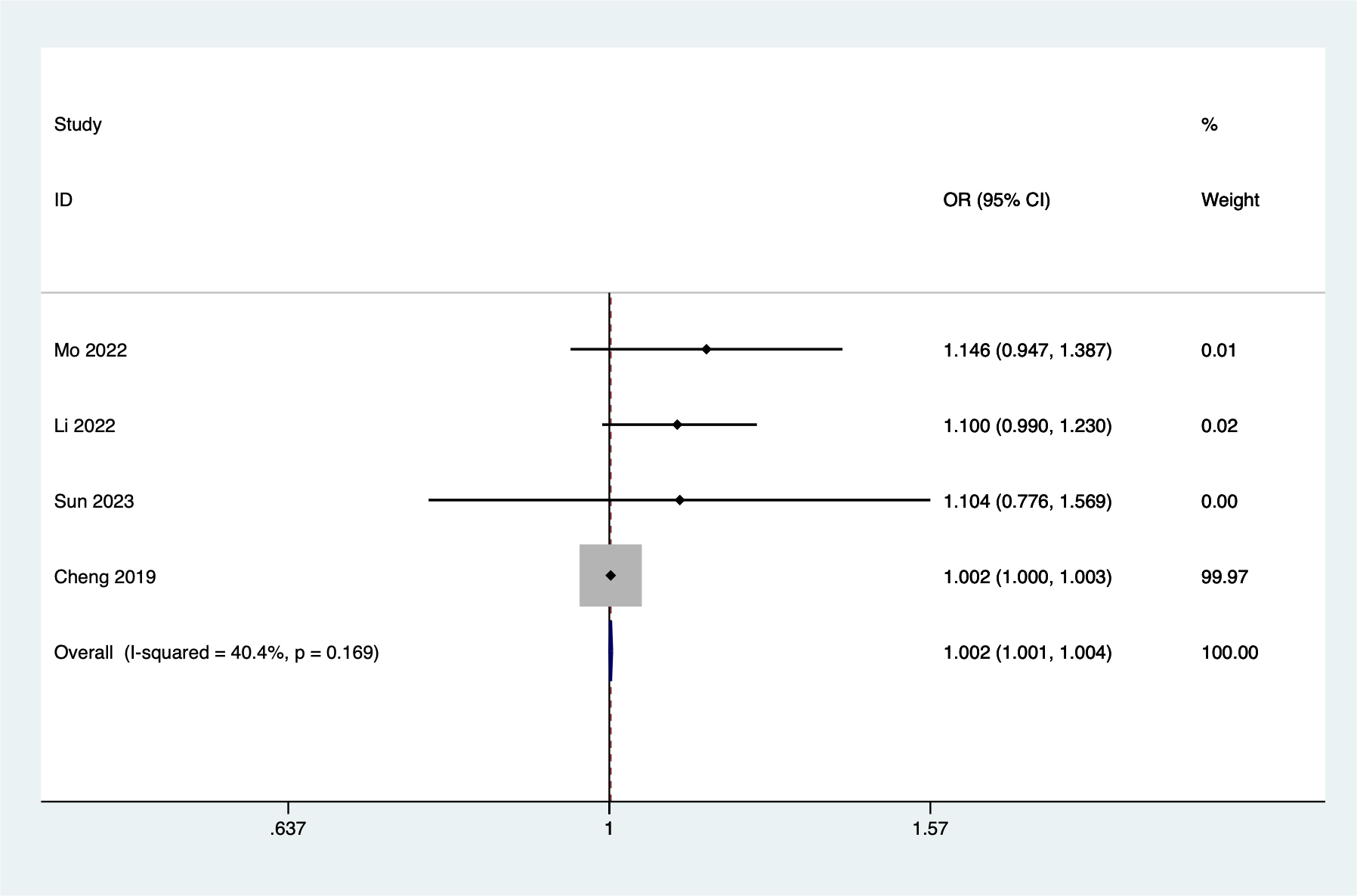

Supplement: Supplementary file 18 — Supporting information. [file CLC-47-e24238-s004.tif]

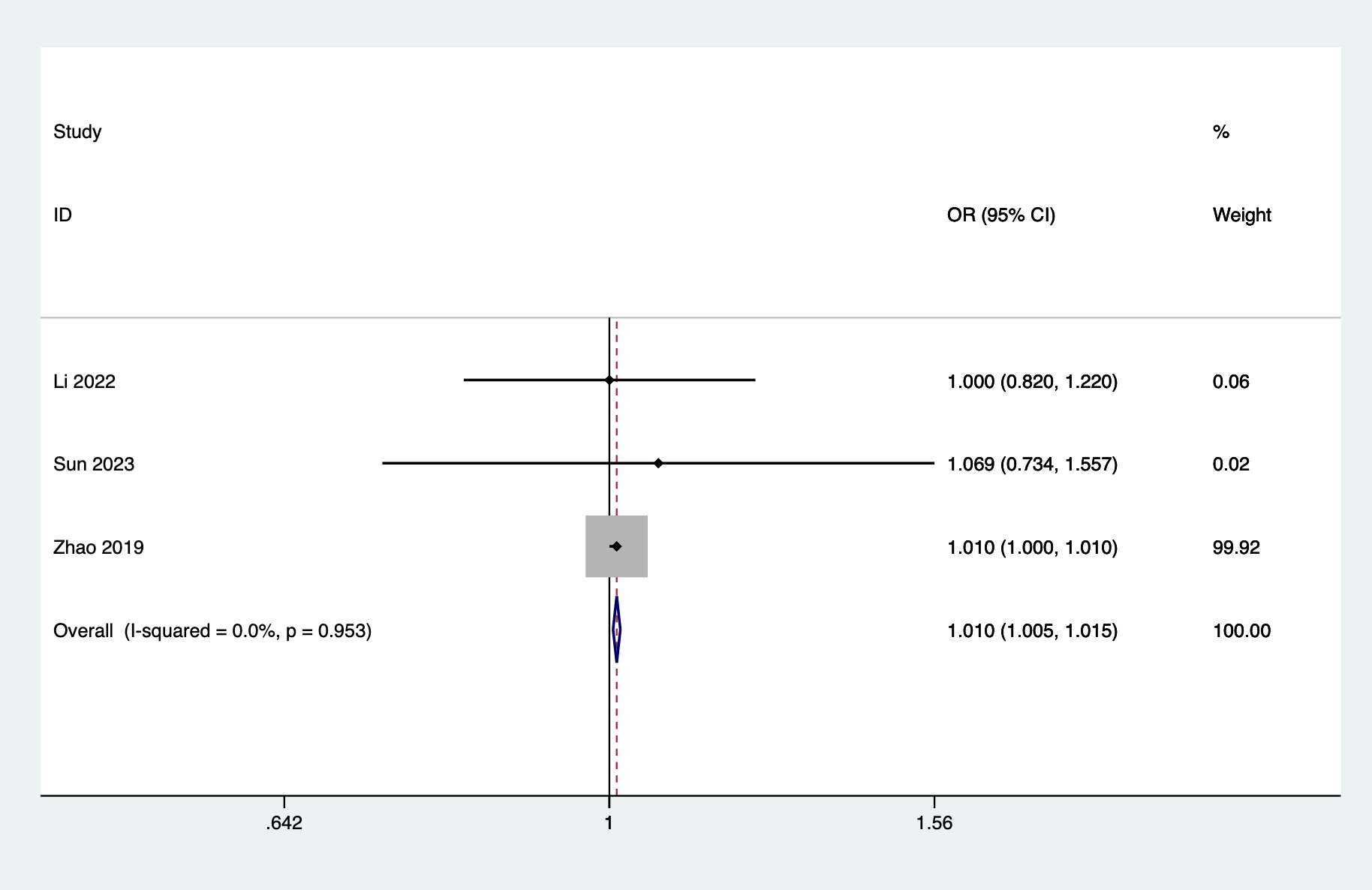

Supplement: Supplementary file 20 — Supporting information. [file CLC-47-e24238-s015.tif]

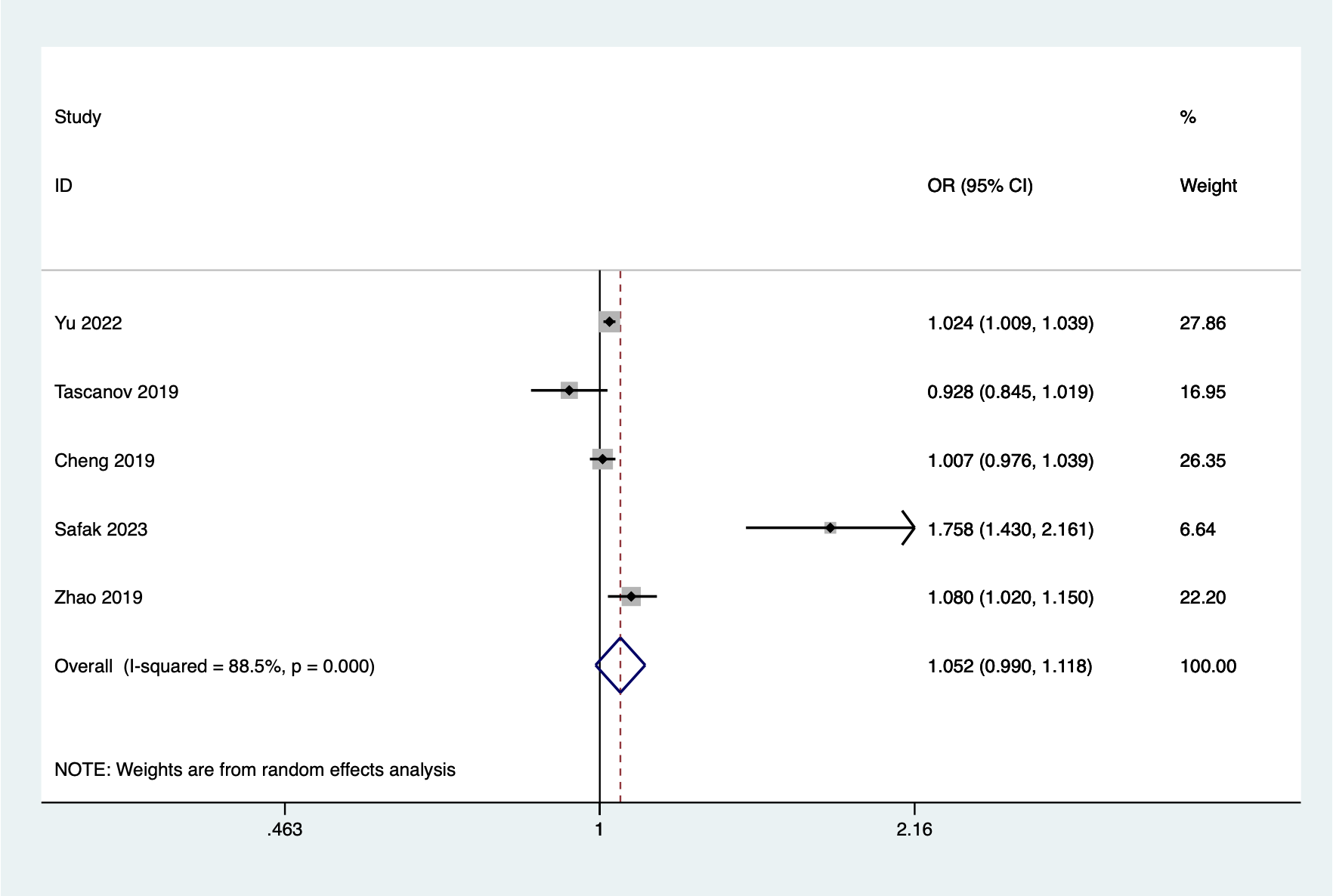

Supplement: Supplementary file 22 — Supporting information. [file CLC-47-e24238-s008.tif]

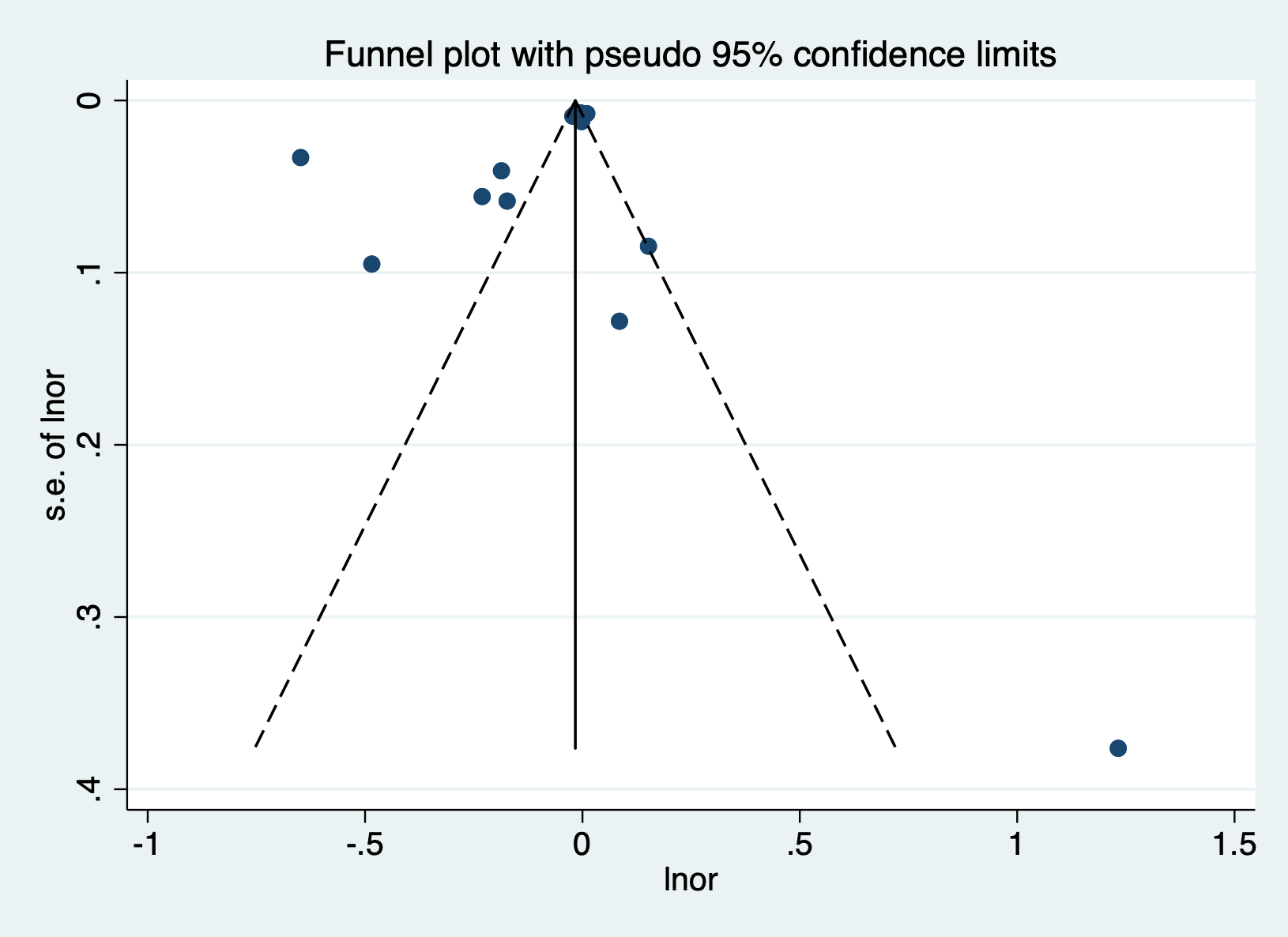

Supplement: Supplementary file 24 — Supporting information. [file CLC-47-e24238-s003.tif]
